# The gut microbiota in colorectal cancer: role in cytokine regulation, intestinal immune barrier dysfunction

**DOI:** 10.3389/fonc.2026.1866422

**Published:** 2026-07-01

**Authors:** Pengfei Zhou, Youcheng He, Haitao Qu, Yanyang Zhou, Hui Pan

**Affiliations:** 1 Anorectal Department, The First Affiliated Hospital of Henan University of CM, Zhengzhou, Henan, China; 2 Department of Gastroenterology, Second People’s Hospital Affiliated to Fujian University of Traditional Chinese Medicine, Fuzhou, Fujian, China

**Keywords:** colorectal cancer, cytokines, gut microbiota, immune barrier, immunotherapy, tumor microenvironment

## Abstract

Colorectal cancer (CRC) remains a leading cause of cancer-related morbidity and mortality worldwide. In addition to genetic susceptibility and environmental exposures, accumulating evidence suggests that the gut microbiota plays a critical role in colorectal carcinogenesis. Dysbiosis of the intestinal microbiota can reshape both local and systemic immune landscapes through persistent inflammatory signaling, aberrant cytokine production, and disruption of the intestinal immune barrier. These microbiota-driven alterations contribute to epithelial injury, chronic inflammation, tumor initiation, and disease progression. Recent advances have highlighted complex interactions among gut microorganisms, cytokine networks, and immune cells within the tumor microenvironment, as well as their influence on responses to anticancer therapies. Microbial metabolites and pathogen-associated molecular patterns can activate key signaling pathways, including nuclear factor-κB and signal transducer and activator of transcription 3, thereby modulating immune surveillance and tumor-promoting inflammation. However, most current evidence is derived from preclinical models, and the causal relationships between specific microbial signatures and CRC remain to be fully established. In this review, we provide a comprehensive and critical overview of current evidence on the role of the gut microbiota in CRC, with a particular focus on microbiota-mediated regulation of cytokines and the intestinal immune barrier. We further discuss the mechanistic links between dysbiosis, immune dysregulation, and colorectal tumorigenesis, and highlight emerging therapeutic strategies targeting the microbiota–immune axis. A better understanding of these interactions may facilitate the development of microbiota-based diagnostic and therapeutic approaches for CRC.

## Introduction

Colorectal cancer (CRC) is one of the most common malignancies worldwide and exhibits a unique characteristic in its close relationship with the gut microbiota (1), which constitutes an important component of the tumor microenvironment. Through the release of various metabolites, proteins, and macromolecules, the gut microbiota interacts with colonic epithelial cells and host immune cells, thereby influencing CRC progression. As research on the gut microbiota has advanced, its role in disease treatment has become increasingly clear (2). However, it remains incompletely understood whether gut microbiota alterations are a direct cause of CRC or one of several contributing factors, and whether modulation of the gut microbiota can be exploited therapeutically.

Gut microorganisms affect numerous physiological processes, including digestion, metabolism, and immune function (3). They can also contribute to carcinogenesis. The microbiota has been proposed as a contributing factor to cancer hallmarks, given its dynamic interactions with systemic immune components, inflammatory markers, and tumor immunity (4). Gut microorganisms can induce ecological changes by influencing other microbes (5), affect autophagy and apoptosis in intestinal epithelial cells (6), modulate intestinal-associated lymphoid tissues (7), and elicit immune responses against microbial antigens that cross-react with tumor-associated antigens (8).

Under normal conditions, the gut microbiota exists in a dynamic balance. When external pathogenic factors disturb its composition or abundance, various diseases may ensue. The gut microbiota not only participates in host metabolism but also co-varies with host gene expression (9, 10). Elucidating the interplay between microbiota changes and host gene function is of great significance for understanding the genetic basis of diseases, including CRC.

In this review, we provide a comprehensive overview of current evidence on the role of the gut microbiota in CRC, with a particular focus on microbiota-mediated regulation of cytokines and the intestinal immune barrier. We further discuss the mechanistic links between dysbiosis, immune dysregulation, and colorectal tumorigenesis, and highlight emerging therapeutic strategies targeting the microbiota–immune axis.

## The gut microbiota in humans

The gut microbiota refers to the community of microorganisms inhabiting the intestines, which is closely related to host nutrition, immunity, and metabolism. The colon harbors the most diverse microbial population, and due to its large number of microorganisms and its close relationship with human health, the gut microbiota is often referred to as the “second genome” of humans and is considered the largest virtual organ of the human body, influencing the digestive, circulatory, immune, and nervous systems. Among all microbial communities colonizing the human body, the gut microbiota exhibits the greatest stability (11).

Changes in gut microbiota diversity are strongly linked to many diseases, including tumors, digestive disorders, diabetes, and cardiovascular diseases (12). The composition and distribution of the gut microbiota are affected by various diseases and environmental factors; in turn, the microbiota can influence the host’s pathophysiological state by regulating the immune system, directly acting on target organs and tissues, or through its metabolites (13). The normal gut microbiota effectively inhibits pathogenic bacteria and helps prevent intestinal dysfunction, primarily through interbacterial interactions that induce host immune-inflammatory responses. Fecal microbiota transplantation has shown therapeutic potential in treating intestinal diseases, including ulcerative colitis and Crohn’s disease (14) (15) (16).

The gastrointestinal tract is the most intensively studied microbial ecosystem, characterized by wide distribution and high microbial diversity. Diversity varies along the length of the gut and spatially between the mucosa and the intestinal lumen, influenced by environmental factors such as pH, bile acid concentration, digestive retention time, mucosal properties, and host defense factors (17) (18). Quantitatively, the gastrointestinal tract is the most densely colonized surface in the human body, with a bacterial load of approximately 10¹^4^ CFU/g. Common genera include *Bacteroides*, *Enterococcus*, and *Bifidobacterium*. Environmental factors, diet, antibiotic use, chemotherapy, gastrointestinal infections, and changes in host immune status can transiently or permanently alter the intestinal ecosystem, leading to dysbiosis, which manifests as a decrease in beneficial bacteria and overgrowth or population shifts of other commensals. Dysbiosis can affect health through the growth of opportunistic pathogens, alteration of host metabolic profiles, and increased inflammation (19).

Most of the body’s microbiota colonizes the gut, and the relationship between gut microbiota balance, dysbiosis, and digestive disorders is well established. Patients with Crohn’s disease (CD) exhibit altered gut microbiota diversity, and the gut microbiota has important implications for CD prevention, treatment, and prognosis (20). Similarly, gut microbiota alterations are associated with other gastrointestinal diseases, including cirrhosis, ulcerative colitis, and irritable bowel syndrome. These findings indicate that changes in gut bacteria may influence the pathogenesis of inflammatory bowel disease (IBD). In addition to environmental, dietary, and pharmacological factors, host genetics can influence the gut microbiota. Studies have shown that human genetics explains 1.9%–8.1% of the variation in gut microbiome composition (21), and the associated genetic loci are enriched in genes involved in gut mucosal barrier function, immune response, and food metabolism (22). Nevertheless, these findings require further validation due to the influence of data processing methods and environmental factors (23). Interestingly, infants with similar bifidobacterial composition can have different outcomes depending on their living environment, suggesting that lifestyle is an important determinant of microbial composition (24).

## Gut microbiota metabolites

Metabolites of the gut microbiota, such as trimethylamine N-oxide (TMAO) and short-chain fatty acids (SCFAs), may affect host functions by influencing intestinal barrier function, autonomic activity, chronic inflammatory responses, glucose metabolism, and lipid metabolism (25). Gastrointestinal motility requires complex coordination among neurons, Cajal interstitial cells, immune cells, and smooth muscle to digest and absorb nutrients. Altered gastrointestinal motility is a hallmark of IBS, and the gut microbiota and its metabolites can influence motility through enteric neurons, glial cells, and intestinal myenteric macrophages. At the same time, the gut microbiota and its products influence the development and maturation of enteric glial cells (26).

**Figure 1:**
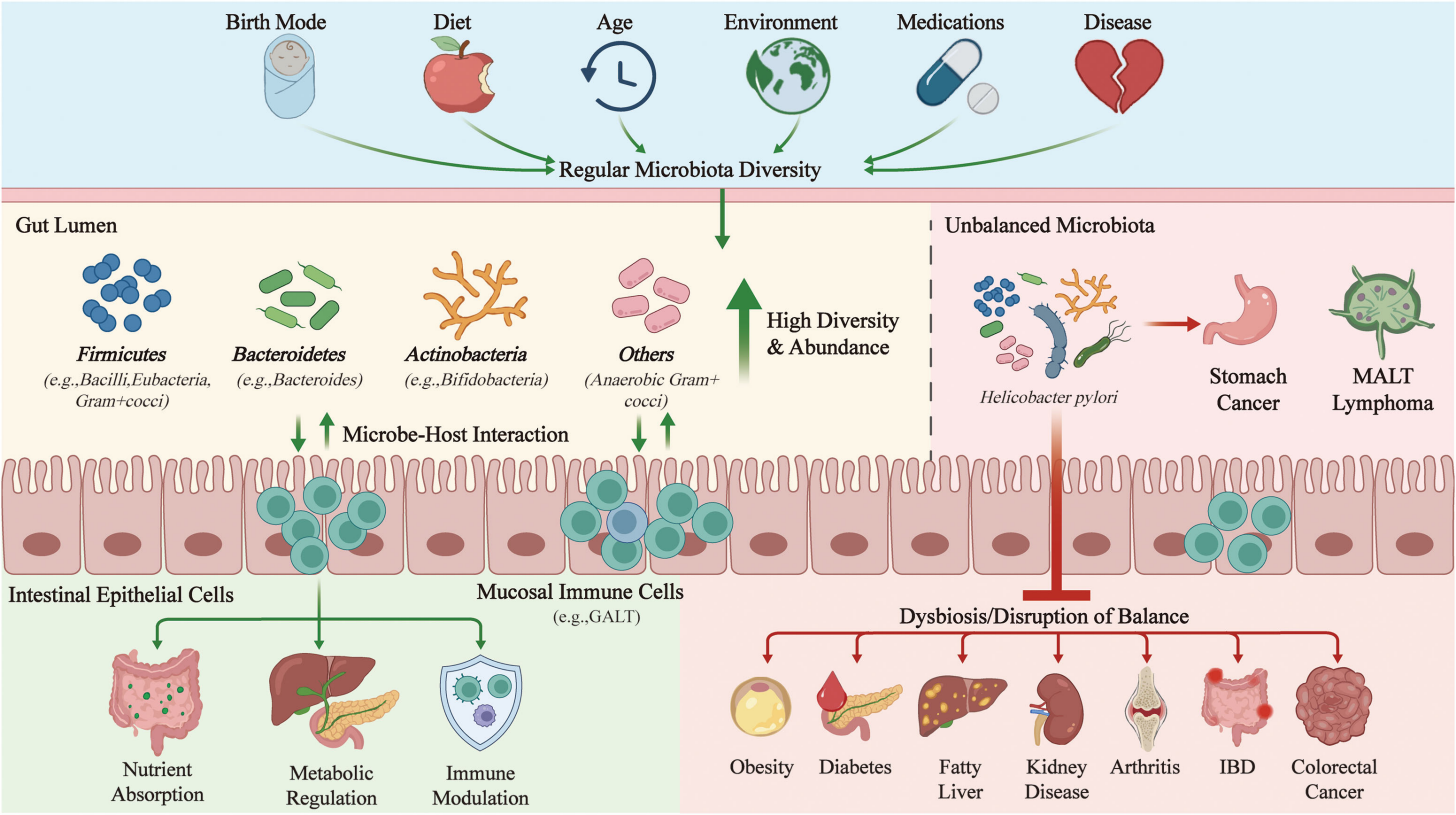
Distribution, biostratum, and function of gut microbiota. The gastrointestinal tract harbors a diverse microbial community that varies along its length and between the mucosa and lumen. The gut microbiota participates in nutrient metabolism, vitamin synthesis, immune regulation, and maintenance of intestinal homeostasis. (Created with https://BioGDP.com).

The gut microbiota can directly induce tumors in the digestive and hematological systems through chronic inflammation or genotoxicity. Gut microbiota imbalance and bacterial translocation can promote tumor cell proliferation and invasion. Therefore, the gut microbiota may serve as a novel tumor biomarker for prognosis assessment and as a therapeutic target (27). The gut microbiota, intestinal epithelial cells, and the human immune system are closely interconnected, and their interactions and balance are closely related to cancer. The gut microbiota can both promote and inhibit cancer. Some probiotics, including yeast probiotics, exert antitumor effects through anti-inflammatory activity, inhibition of malignant proliferation, and inactivation of dietary carcinogens (**Figure 1**).

## Gut microbiota and its composition

The gut microbiota is influenced by factors such as mode of delivery, diet, age, environment, medications, and disease type. High-throughput sequencing-based metagenomics enables the identification and classification of bacteria (28). In healthy adults, the most predominant gut microbiota phyla are *Firmicutes* and *Bacteroidetes*, followed by *Actinobacteria*, *Proteobacteria*, and *Verrucomicrobia* (29). Most gut microorganisms are obligate anaerobes, with bacilli being the most abundant, including *Bacteroides*, *Eubacterium*, *Bifidobacterium*, and *anaerobic Gram-positive cocci* (30). The composition of the gut microbiota is highly complex, with extreme diversity and individual differences at the species and even strain levels. The species and relative abundance of the gut microbiota in healthy adults are similar at the phylum level, but the microbiota varies within the intestinal lumen and within tissues; the biological significance of this variation remains unknown (31).

The gut microbiota is involved in many important physiological functions, including nutrition, metabolism, and immunity, and plays an essential role in maintaining intestinal homeostasis. Under normal physiological conditions, the gut microbiota coexists with the host in a state of dynamic equilibrium; once this equilibrium is disrupted, disease may occur (32). Many diseases have been linked to the gut microbiota, including obesity, fatty liver disease, diabetes, kidney disease, arthritis, IBD, and CRC. Among the studies on the relationship between the gut microbiota and tumors, the most definitive is the association between Helicobacter pylori infection and gastric cancer and mucosa-associated lymphoid tissue (MALT) lymphoma. However, the precise relationship between the gut microbiota and CRC has not been fully elucidated.

## Microbiota-driven cytokine networks in CRC

The gut microbiota regulates colorectal carcinogenesis through the coordinated action of pro-inflammatory and anti-inflammatory cytokine networks. Under homeostatic conditions, commensal bacteria promote the production of anti-inflammatory cytokines such as IL-10 via regulatory T cell (Treg) differentiation, maintaining immune tolerance and limiting tissue damage (33). Specific bacteria, including *Bacteroides fragilis*, *Bifidobacterium infantis*, and *Clostridium species*, stimulate Tregs to secrete IL-10, which suppresses excessive activation of Th1 and Th17 cells. However, in CRC-associated dysbiosis (characterized by an expansion of pathobionts such as *Fusobacterium nucleatum*, *Escherichia coli*, and *enterotoxigenic Bacteroides fragilis*) the balance shifts toward pro-inflammatory pathways. Microbial products (LPS, flagellin, CpG DNA) activate Toll-like receptors (TLRs) on intestinal epithelial cells and macrophages, triggering NF-κB and STAT3 signaling, which drive the production of IL-6, TNF-α, and IL-1β. These cytokines promote tumor cell survival through upregulation of anti-apoptotic genes (Bcl-2, Bcl-xL, Mcl-1) and create a chronically inflamed microenvironment conducive to tumor initiation and progression (34).

IL-17 and IL-22, primarily produced by Th17 cells and type 3 innate lymphoid cells (ILC3s). Dysbiosis promotes Th17 differentiation, and elevated IL-17 levels in CRC tissues correlate with poor prognosis. IL-17 recruits neutrophils, activates NF-κB, and induces angiogenic factors, fostering tumor growth. However, the role of IL-17 in CRC is controversial. While several studies report that IL-17 promotes tumor growth through NF-κB activation and neutrophil recruitment, others have found that IL-17 is required for anti-tumor immunity by recruiting cytotoxic CD8^+^ T cells. These discrepant findings may be explained by differences in tumor stage, the cellular source of IL-17, and the composition of the local microbiota. IL-22 plays a dual role: it supports epithelial barrier repair under acute conditions but, in the setting of chronic inflammation, drives epithelial hyperproliferation and STAT3 activation, promoting tumorigenesis. Meanwhile, anti-inflammatory signals are often compromised in CRC. Butyrate-producing bacteria (e.g., *Faecalibacterium prausnitzii*, *Roseburia*) are depleted in CRC patients, reducing HDAC inhibition-mediated upregulation of anti-inflammatory pathways (35). The resulting imbalance (excess of IL-6, TNF-α, IL-17, and IL-22 over IL-10 and TGF-β) creates a tumor-promoting cytokine milieu. Understanding these networks provides opportunities for therapeutic intervention, including IL-6/STAT3 inhibitors and microbiota-based strategies to restore cytokine balance.

**Figure 2:**
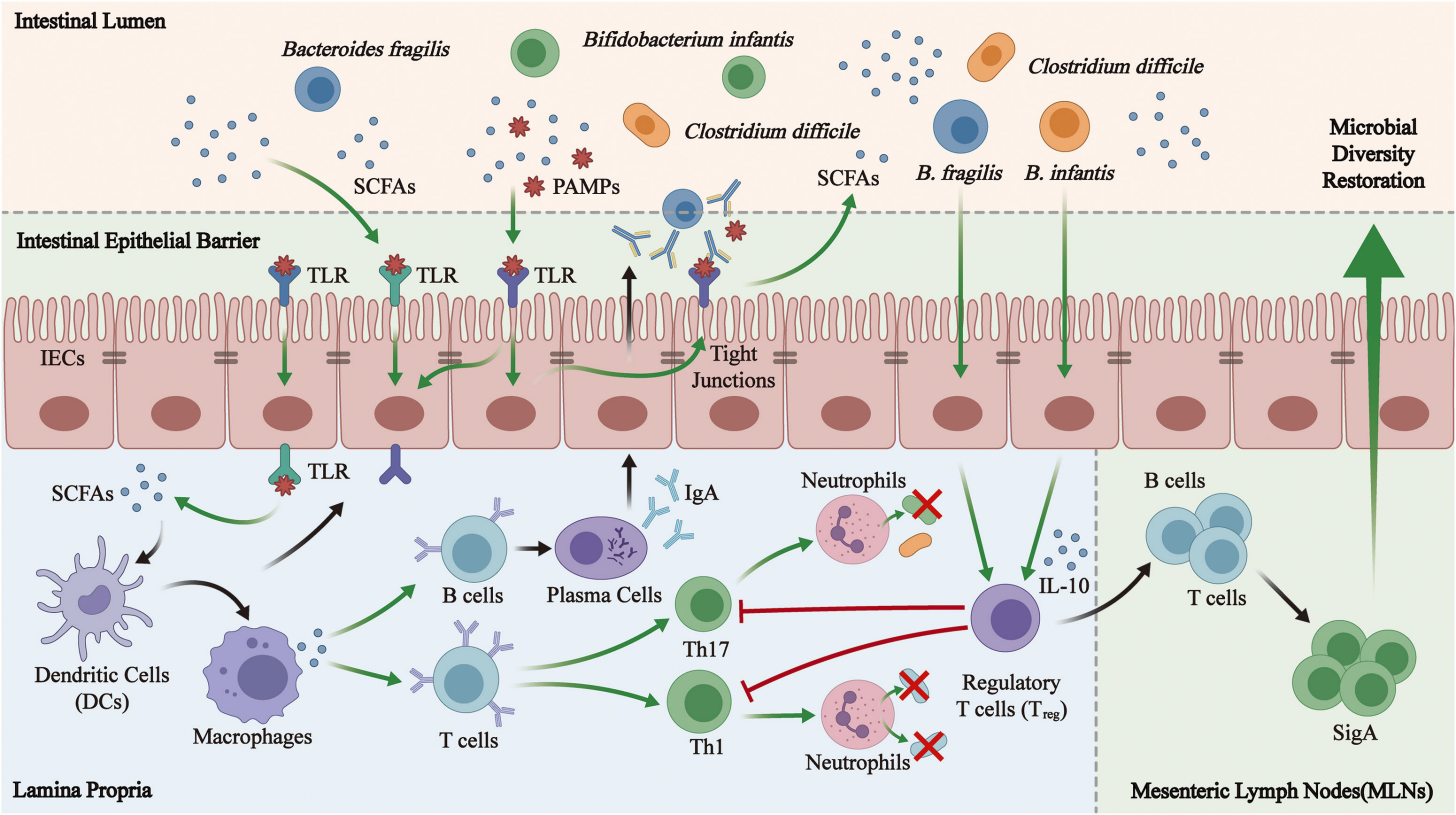
Microbiota-driven regulation of cytokine networks and the intestinal immune barrier in colorectal cancer. Gut microbiota regulates host mucosal immune function through TLR signaling, immune cell differentiation, and metabolite production. Under homeostatic conditions, commensal bacteria (e.g., *B. fragilis*, *Clostridium*) promote Treg differentiation and IL-10 production, while butyrate inhibits HDAC to exert anti-inflammatory effects. Intestinal bacteria are phagocytosed by DCs and macrophages, leading to B cell maturation and IgA secretion in the intestinal lumen, as well as T cell differentiation into Th17 and Th1 cells. TLR activation on IECs and immune cells triggers signaling cascades that enhance cellular barriers and stimulate lamina propria immune cells. This integrated regulation maintains intestinal homeostasis. In CRC, dysbiosis disrupts this balance, leading to chronic inflammation and tumor promotion.

It is also important to clarify the mechanism by which short-chain fatty acids (SCFAs), particularly butyrate, exert their anti-inflammatory effects. Butyrate through inhibition of histone deacetylases (HDACs), which reduces NF-κB transcriptional activity; and activation of G-protein-coupled receptors such as GPR109A, which downstream suppresses NF-κB activation and promotes anti-inflammatory responses (36). This correct understanding is essential for interpreting the immunomodulatory role of butyrate-producing bacteria in CRC (**Figure 2**).

Most studies on cytokine networks in CRC are derived from preclinical models or small human cohorts with limited statistical power. The relative contributions of specific cytokines to CRC progression in humans remain unclear, and therapeutic targeting of cytokines (e.g., IL-6/STAT3 inhibitors) has not yet been validated in CRC-specific clinical trials. Furthermore, the complex crosstalk among cytokines (including both synergistic and antagonistic interactions) is often simplified in experimental systems, potentially missing emergent properties of the network.

## The intestinal immune barrier: microbiota interactions and CRC

The intestinal immune barrier comprises physical, immune, and microbiological layers that separate the luminal microbiota from host tissues. The physical barrier includes the mucus layer (primarily MUC2) and tight junction proteins (occludin, claudins, ZO-1) between intestinal epithelial cells (IECs). The immune barrier features secretory IgA (sIgA), produced by lamina propria plasma cells, and intraepithelial lymphocytes. The gut microbiota critically regulates barrier integrity. Beneficial commensals reinforce barrier function through butyrate production, which inhibits histone deacetylases (HDACs), upregulates tight junction expression, and promotes MUC2 synthesis (35). Additionally, specific bacteria (e.g., B. fragilis, Clostridium clusters) enhance sIgA secretion and Treg-mediated IL-10 production, which suppresses inflammation that would otherwise damage the barrier (37) (38). Conversely, CRC-associated pathobionts directly disrupt barrier integrity: *B. fragilis toxin* (BFT) cleaves E-cadherin, *F. nucleatum* FadA binds E-cadherin and activates β-catenin signaling, and mucin-degrading bacteria (e.g., *Ruminococcus gnavus*) thin the protective mucus layer.

When barrier integrity is compromised, luminal microbial products—LPS, flagellin, CpG DNA—translocate into the lamina propria, where they activate TLRs on IECs and macrophages, triggering NF-κB and STAT3 signaling and subsequent production of pro-inflammatory cytokines (IL-6, TNF-α, IL-1β, IL-17). This chronic inflammation establishes a positive feedback loop: inflammation-induced barrier disruption allows further bacterial translocation, amplifying cytokine production and perpetuating tissue injury. In the context of CRC, this cycle promotes epithelial damage, compensatory proliferation, DNA damage (including from genotoxins such as *E. coli colibactin*), and tumor progression. Strategies to restore barrier function—including probiotics (e.g., *Lactobacillus*, *Bifidobacterium*), butyrate supplementation, and potentially fecal microbiota transplantation—are being explored for CRC prevention and treatment, though clinical evidence remains preliminary. The close relationship between barrier dysfunction, microbial translocation, and inflammation suggests that barrier integrity could serve as both a biomarker for CRC risk and a therapeutic target.

Currently, direct evidence linking increased intestinal permeability to CRC development in humans is limited. Most studies rely on serum markers of translocation (e.g., LPS-binding protein) rather than direct measurement of barrier function. Whether barrier dysfunction is a cause or a consequence of CRC remains unresolved, and prospective studies are needed to establish temporality. Despite these limitations, the barrier-microbiota-inflammation axis has been proposed as a promising source of early diagnostic biomarkers and therapeutic targets for CRC (39).

## Gut microbiota dysbiosis in colorectal cancer

In the long-term evolutionary process, different genera and species of gut bacteria and the human body have maintained a state of dynamic equilibrium, forming mutually constraining and counterbalancing relationships. Gut bacteria play important roles in nutrient metabolism and uptake, maintenance of the intestinal mucosal barrier, and regulation of the intestinal mucosa. Under normal conditions, the human body maintains a relatively stable microbial structure. However, factors such as diet or drugs can alter the microbial structure, disrupt the host intestinal mucosal barrier, and induce a persistent chronic inflammatory state of the mucosa. This prompts intestinal epithelial cells to undergo a sequence of injury, proliferation, epithelial atypia, and adenoma formation, ultimately leading to CRC.

CRC is associated with various intestinal bacteria, including *Escherichia coli*, *Bacteroides fragilis*, *Fusobacterium nucleatum*, and certain anaerobic streptococci, which may induce cancer through the release of genotoxins (40). When CRC undergoes distant metastasis, *Fusobacterium*, *Bacteroides*, and Proteus species present in primary CRC tissues metastasize along with the tumor, suggesting a correlation between the local microbiota of primary tumors and metastatic tumor tissues (41). *Fusobacterium nucleatum* is enriched in CRC tissues, as consistently reported across multiple case-control studies (42). However, most of these studies are cross-sectional, precluding determination of whether *F. nucleatum* enrichment precedes or follows tumor development. Longitudinal studies in at-risk populations are needed to establish temporality. When CRC metastasizes distantly, certain bacterial genera in human CRC tissues also metastasize with the tumor, indicating stability of the microbial composition between the primary tumor and metastases. For example, mice transplanted with human primary CRC feces retained *F. nucleatum* activity through successive generations, whereas mice treated with the antibiotic metronidazole showed decreased abundance of *F. nucleatum*, reduced cancer cell proliferation, and inhibited tumor growth. These findings demonstrate for the first time that gut microorganisms can assist in cancer metastasis and help cancer cells colonize other locations in the body, providing new insights for cancer diagnosis and treatment (43).

**Figure 3:**
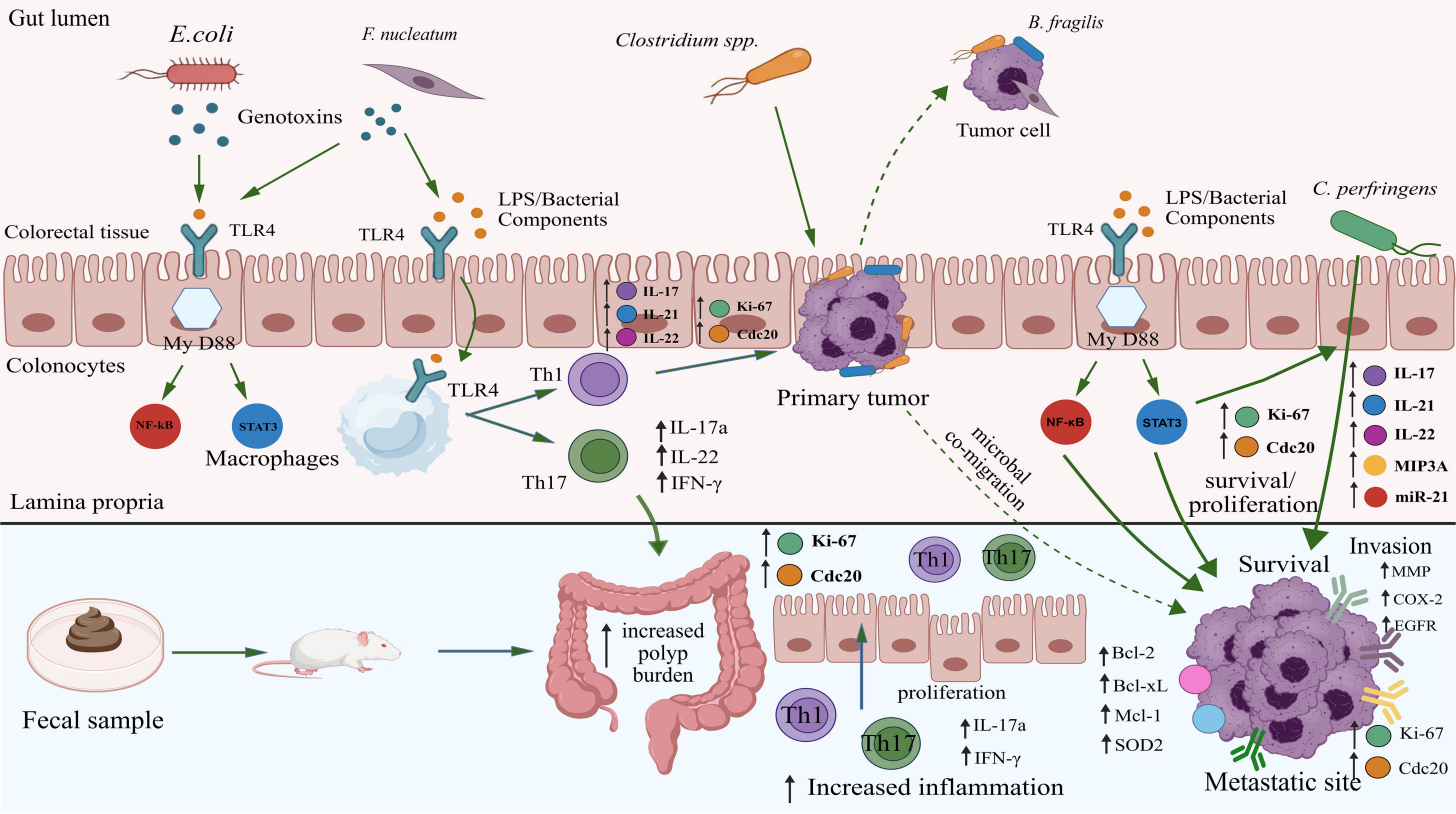
Disruption of gut microbiota and incidence of colorectal cancer. Dysbiosis disrupts the intestinal mucosal barrier, induces chronic inflammation, and promotes bacterial translocation. Microbial products activate TLR4 on intestinal epithelial cells and macrophages, triggering downstream NF-κB and STAT3 signaling. This upregulates proto-oncogenes (Bcl-2, Bcl-xL, Mcl-1, SOD2) and inflammatory factors (IL-17a, IL-22, IFN-γ), promoting intestinal epithelial cell proliferation, epithelial atypia, adenoma formation, and ultimately colorectal cancer.

Although the precise mechanisms of microbiota-induced carcinogenesis remain unclear, a growing body of research suggests that chronic inflammation of the intestinal mucosa driven by the gut microbiota underlies CRC development. In a landmark study, gnotobiotic mice colonized with feces from CRC patients developed significantly more colonic polyps than mice receiving healthy donor feces (44). While this finding strongly supports a causal contribution of the microbiota to tumor promotion, it is important to note that these experiments were conducted in mice housed under specific pathogen-free conditions, which do not fully recapitulate the complex microbial and immune environment of humans. Furthermore, the translatability of such findings is limited by inter-individual variability in human microbiota composition and the polygenic nature of CRC susceptibility. At the cellular and genetic levels, CRC-derived fecal bacteria promoted proliferation of intestinal mucosal epithelial cells and infiltration of Th1 and Th17 immune cells. Transcriptional levels of the inflammatory factors IL-17a, IL-22, and IFN-γ, as well as the proto-oncogenes Ki-67 and Cdc20, were significantly increased in these mice, confirming that intestinal bacteria-induced mucosal inflammation plays a role in CRC pathogenesis (44). Several inflammatory signaling pathways mediate the bridge between pathogen-triggered inflammatory responses and cancer. The intestinal epithelial cell and macrophage surface receptor TLR4, as well as the downstream transcription factors NF-κB and STAT3, play key roles (45). Overexpression of TLR4 on the surface of colon cancer epithelial cells enhances the effect of bacterial lipopolysaccharide (LPS) on the inflammatory microenvironment and promotes the secretion of matrix metalloproteinases (MMPs), cyclooxygenase-2 (COX-2), and epidermal growth factor receptor (EGFR), thereby increasing tumor cell adhesion and invasion. Activation of NF-κB and STAT3 upregulates the expression of the proto-oncogenes Bcl-2, Bcl-xL, Mcl-1, and SOD2, promoting tumor cell proliferation and survival (46) (**Figure 3**).

A large body of correlative evidence links gut microbiota dysbiosis to CRC. However, establishing causality in humans remains challenging due to several factors. First, most human studies are cross-sectional, making it impossible to distinguish whether dysbiosis is a cause or a consequence of CRC. Second, the gut microbiota is highly variable across individuals and influenced by numerous confounders (diet, medications, genetics). Third, findings from animal models, while supportive of causality, may not fully translate to humans due to differences in microbiota composition, immune system development, and lifespan. Therefore, while the weight of evidence suggests that dysbiosis contributes to colorectal carcinogenesis, definitive causal proof in humans would require large-scale prospective cohort studies with pre-diagnostic microbiome sampling, which are currently lacking.

## Therapeutic implications and future directions

The gut microbiota has emerged as a critical determinant of response to immune checkpoint inhibitors (ICIs), including anti-PD-1/PD-L1 and anti-CTLA-4 antibodies, in multiple cancer types (47). In CRC, particularly in patients with mismatch repair-deficient (dMMR) tumors who are eligible for ICI therapy, accumulating evidence suggests that specific microbial signatures correlate with treatment outcomes (48). For example, enrichment of *Akkermansia muciniphila*, *Bifidobacterium species*, and *Faecalibacterium prausnitzii* has been associated with favorable ICI responses in melanoma and non-small cell lung cancer, and emerging data suggest similar associations in dMMR CRC (49). Conversely, the presence of certain pathobionts, such as Fusobacterium nucleatum, may promote immune evasion and resistance to immunotherapy (50). Mechanistically, the gut microbiota influences ICI efficacy through multiple pathways: modulation of systemic T cell responses via microbial metabolites (e.g., inosine, short-chain fatty acids); cross-reactivity between microbial antigens and tumor-associated antigens; and regulation of intestinal barrier integrity, which affects systemic exposure to microbial products that shape immune tone. However, most of these findings are derived from preclinical models or retrospective human cohorts, and prospective trials evaluating microbiome modulation as an adjunct to ICI therapy in CRC are urgently needed. There is research demonstrated that the gut microbiota significantly influences cancer immunotherapy efficacy by modulating immune responses, remodeling the tumor microenvironment, and producing key metabolites, with strategies such as fecal microbiota transplantation and probiotics showing promise in enhancing responses to ICIs (51). Xie et al. further highlighted that altered gut microbiota enriched in pathobionts can actively promote immune evasion and disrupt anti-tumor immunity, while beneficial commensals such as *Lactobacillus* and *Bifidobacterium* species are emerging as therapeutic probiotics for cancer prevention and as adjuvants for cancer therapy (52).

Recent advances in multi-omics technologies are transforming the study of host-microbiome-immune crosstalk by enabling comprehensive profiling of the gut ecosystem at multiple molecular layers (53). Metagenomics provides strain-level resolution and functional gene profiling, allowing identification of specific microbial genes (e.g., biosynthetic gene clusters for genotoxins or immunomodulatory metabolites) associated with CRC (54). Metatranscriptomics reveals which microbial genes are actively expressed, offering insights into real-time microbial activity rather than mere presence. Metabolomics profiles the repertoire of microbial and host-derived metabolites (such as short-chain fatty acids, bile acids, and tryptophan catabolites) that mediate microbiota-immune interactions (53). Single-cell sequencing (e.g., scRNA-seq of immune and epithelial cells from CRC tissues) enables mapping of cellular heterogeneity and ligand-receptor interactions within the tumor microenvironment, which can be integrated with microbiome data to infer causal networks (54). Collectively, these multi-omics approaches hold promise for dissecting the complex mechanisms by which the gut microbiota influences CRC pathogenesis and treatment response.

The integration of multi-omics data with clinical outcomes is paving the way toward precision medicine through microbiota-based biomarkers (55). Specific microbial signatures, such as the relative abundance of *Fusobacterium nucleatum*, *Bacteroides fragilis*, and butyrate-producing bacteria, have been proposed as adjunctive biomarkers for early CRC detection, though sensitivity and specificity remain suboptimal for standalone clinical use (56). In the context of immunotherapy, fecal microbial profiling may eventually help stratify dMMR CRC patients into likely responders versus non-responders, enabling personalized treatment decisions (57) (58). Machine learning models integrating gut microbiome data have also shown promise in predicting clinical outcomes such as carcinoembryonic antigen levels and peripheral nerve invasion status in CRC patients (59). However, several hurdles remain: lack of standardized protocols for sample collection, sequencing, and data analysis; high inter-individual and inter-population variability in gut microbiota composition; the need for large, prospective validation cohorts; and the transition from correlative associations to causally validated biomarkers. Addressing these challenges will require interdisciplinary collaboration among microbiologists, immunologists, computational biologists, and clinicians, as well as the establishment of large-scale biorepositories with linked clinical and multi-omics data.

Although many studies have demonstrated a close relationship between the gut microbiota and CRC development, the structure of the human gut microbiota is vast, and the mechanisms of action of many potential pathogenic bacteria in CRC have not been fully elucidated. Research on the impact of the gut microbiota on intestinal barrier function has tended to focus on individual bacteria, receptors, and molecules. However, the relationship between the gut microbiota and the host is bidirectional (60). The intestinal mucosa regulates gut microbiota structure through mucosal immunity or the production of extracellular vesicles, and different bacterial species coexist or exclude each other in a network. Combining modern biological approaches can help study the complex interactions between the gut microbiota and the host and identify new pathogenic or probiotic bacterial species, thereby providing new technologies and ideas for CRC prevention, diagnosis, and treatment.

Based on the significant effects of probiotics on chemotherapy and immunotherapy observed in animal experiments, the use of probiotics to treat diseases has broad application prospects in gut microbiota research. Although it is not easy to modify the complex intestinal ecosystem with a single bacterial species, fecal microbiota transplantation from healthy donors has achieved good results in treating Clostridium difficile infection and IBD, providing a new therapeutic target and direction for cancer treatment (61).

Most current studies are based on animal experiments or sporadic CRC (62). Escherichia coli and Bacteroides fragilis have been found in patients with hereditary CRC, where they collaborate to promote CRC development, and similar gut microbiota have been found in sporadic CRC (63). The relationship between the gut microbiota and tumor development, as well as its potential application in cancer therapy, is receiving increasing attention. However, how environmental factors alter the species and abundance of the gut microbiota, and the specific molecular mechanisms of gut microbiota in CRC development, require further research.

## Conclusion

The gut microbiota can enhance the host’s immune response against tumors and may even determine the efficacy of treatment. Although the role of the gut microbiota should not be underestimated, the clinical antitumor efficacy of combining gut microbiota modulation with immunotherapy remains uncertain due to differences between experimental animals and humans. Nevertheless, various gut bacterial genera are closely related to tumor development. While existing experiments have confirmed the immunomodulatory role of individual gut bacterial genera, they have also presented new challenges: how to identify gut bacterial genera with tumor specificity, whether they can be used as indicators to assess immunotherapy efficacy, and how to fully apply them in clinical practice. In addition, how to select antibiotics for cancer patients and whether antibiotic use negatively impacts cancer treatment are issues that require careful consideration. Therefore, further in-depth research on the gut microbiota and tumor immunotherapy to understand their mechanisms of action, and attempts to combine these two fields to establish novel gut microbiota-based tumor markers, may lead to new tumor-targeted therapeutic strategies. The clinical translation of microbiota-based therapies for CRC faces substantial hurdles. While probiotics and FMT have shown promise in preclinical models and in small human studies for other indications (e.g., C. difficile infection), randomized controlled trials in CRC patients are lacking. Moreover, potential safety concerns (including the risk of bacteremia with live probiotics in immunocompromised patients and the theoretical risk of FMT transmitting unknown pathogens) must be addressed before widespread clinical adoption.
